# Location biases in ecological research on Australian terrestrial reptiles

**DOI:** 10.1038/s41598-020-66719-x

**Published:** 2020-06-16

**Authors:** Renee Louise Piccolo, Jan Warnken, Alienor Louise Marie Chauvenet, James Guy Castley

**Affiliations:** 1 School of Environment and Science, Griffith University, Gold Coast campus, Australia 4222; 2 Environmental Futures Research Institute, Griffith University, Gold Coast campus, Australia 4222; 3 Australian Rivers Institute, Griffith University, Gold Coast campus, Australia 4222

**Keywords:** Biogeography, Conservation biology, Ecological modelling

## Abstract

Understanding geographical biases in ecological research is important for conservation, planning, prioritisation and management. However, conservation efforts may be limited by data availability and poor understanding of the nature of potential spatial bias. We conduct the first continent-wide analysis of spatial bias associated with Australian terrestrial reptile ecological research. To evaluate potential research deficiencies, we used Maxent modelling to predict the distributions of 646 reptile studies published from 1972 to 2017. Based on existing distributions of 1631 individual reptile study locations, reptile species richness, proximity to universities, human footprint and location of protected areas, we found the strongest predictor of reptile research locations was proximity to universities (40.8%). This was followed by species richness (22.9%) and human footprint (20.1%), while protected areas were the weakest predictor (16.2%). These results highlight that research effort is driven largely by accessibility and we consequently identify potential target areas for future research that can be optimised to ensure adequate representation of reptile communities.

## Introduction

Conservation efforts are frequently targeted towards securing protected area networks^1^, and the development of effective conservation instruments requires detailed, spatially explicit and comprehensive data on ecological communities^2,3^. Therefore, understanding spatial bias in ecological information that underpins the planning, design and implementation of biodiversity conservation measures is critical^2–5^. Declining field-based research and a greater reliance on meta-analyses and large datasets for modelling^6^, have revealed that large-scale analyses of biodiversity data (e.g. occurrence records) can be limited by the coverage and adequacy of their response data^7,8^. Therefore, indiscriminate utilisation of accessible datasets without understanding potential sources of bias within them could deliver poor conservation decision-making. Additionally, several taxonomic groups are under-represented in ecological studies, especially where such species are obscure and less charismatic^9^, potentially leading to further bias.

Globally, reptiles have been the subject of substantially less ecological research compared to other vertebrates such as birds and mammals^9,10^. Factors contributing to the relative paucity of reptile research include the difficulty in sampling cryptic reptile species^11^; reptile anti-social behaviours portrayed as less appealing to study^12^; or the fearsome reputation of some reptiles shaped by societal attitudes^13^. Such overall poor appeal can influence prioritisation of species conservation^14^.

Global reptile diversity is the highest of all terrestrial vertebrates^15^, particularly so in Australia^16,17^, yet their under-representation in research has led to major knowledge deficiencies in reptile ecology^10,18^. Existing spatial bias as a result of greater sampling effort in regions of high species richness has been highlighted for work on island reptiles^19^ and vouchered plant specimen^20^. Such focus has generated a concentration of ecological research localities in biodiversity hotspots and tropical regions^5,10^. Preferences for choosing research locations has also been shown to be affected by ease of access^4,8^ and proximity to higher human population density^2,21^. Conscious bias in selecting sampling sites may deliver greater efficiencies where surveys are targeting uncommon species at the local scale^22^, but the effects of such bias may be more noticeable at regional or national scales. Such spatial biases can consequently result in unreliable estimates of population sizes, species richness and community dynamics, or simply result in blank spots, i.e. locations that are poorly studied^3,20^. Furthermore, global conservation assessments such as the IUCN Red List frequently rely on accurate estimates of population sizes, distributions and threats^23,24^. These assessments are impeded by a general lack of knowledge about species, especially rare ones, but also uncertainty and bias in available data^25^, particularly with regards to spatial information^10,18^.

This study presents a first continent-wide investigation of the spatial biases in ecological research on Australian terrestrial reptiles through a systematic review of the literature, coupled with an analysis of survey effort using species distribution modelling (SDM)^26^. Species distribution models are routinely used to model species occurrence data as a function of multiple environmental predictors^27^. Such predictive modelling can also be used to identify factors that influence the spatial distribution of certain elements within a landscape (e.g. conservation lands)^28^. It is therefore possible to empirically assess factors affecting the distribution of research study location distributions, where these were reported accurately, to improve future survey strategies. We hypothesised that accessibility would exert an overriding influence on where research studies had been conducted. Our analysis aimed to: 1) determine the overall spatial pattern of Australian terrestrial reptile ecological research; 2) model the predicted influence of ‘proximity to universities’, ‘human footprint’, ‘species richness’, and ‘protected areas’ on study locations; and 3) recommend target areas for future research priorities in order to address potential spatial bias in Australian reptile research.

## Methods

### Literature search

**Figure 1:**
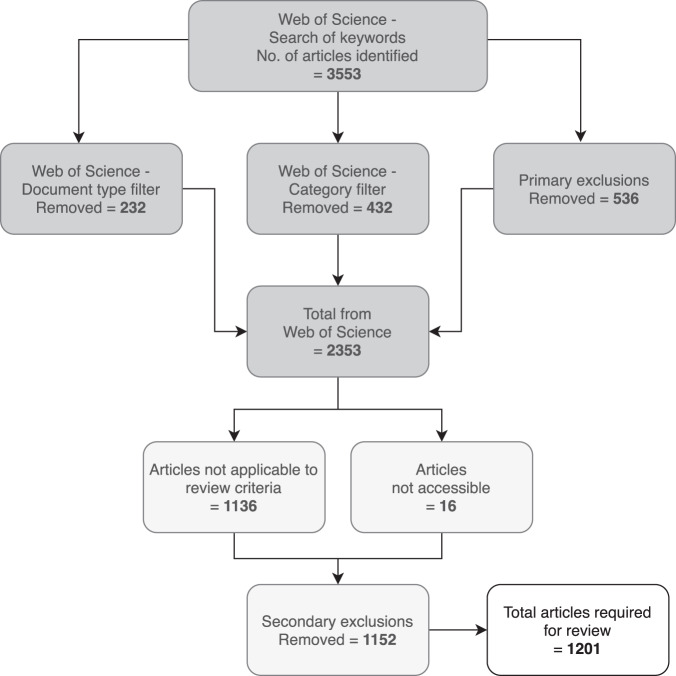
PRISMA flow chart indicating systematic approach and search results for filtering research articles through the Web of Science database.

We completed a systematic quantitative review of peer-reviewed Australian terrestrial reptile research papers published in English using the PRISMA method^29^ to identify, screen and select relevant papers for analysis. Papers published from 1972 to July 2017 were sourced from the Web of Science database using Boolean searches of primary and secondary keywords (Supplementary S1) across all potential research discipline categories (Supplementary S2). All papers identified were screened to exclude those that were not applicable to this study. Exclusion criteria included studies on captive animals, laboratory only studies, other taxa, studies outside of Australia, medical related research, studies on museum specimens only and modelling/desktop analyses on previous studies (n = 1136, Fig. 1). Articles that were inaccessible (n = 16) were also excluded from the review (Fig. 1).

### Database structure

Information from each remaining peer-reviewed article was compiled using two principal metrics characterising (i) each ‘study’ and (ii) its reported ‘research’. ‘Study’ metrics included; the paper’s title, authors’ names, year and journal of publication, the state or territory where the study was conducted, the number of study sites surveyed, and the geographic coordinates of any location information provided by the authors. Those lacking specific location details were also added to this part of the database. ‘Research’ metrics captured the research focus (as primary and sub-categories), methodology used, as well as information about the reptiles studied (Supplementary S3).

### Study location dataset

Spatial analysis included only studies with accurate location information (i.e. geographic coordinates) to maximise the potential of our distribution models for evaluating research location preferences. Coordinates of identified study locations were mapped as a point layer in ArcGIS 10.4™ (ESRI). Where single studies listed multiple sites, each coordinate was treated as an independent study location.

### Predictor datasets

We selected four prominent variables as predictors of reptile study locations in Australia: ‘reptile species richness’, ‘human footprint’, ‘protected areas’ and ‘proximity to universities’. The variables were selected based on previous trends in study location biases of other taxonomic groups^2,4,21^. Other possible environmental predictors that might influence faunal and floral species distributions (e.g. habitat type, climatic zones, local abiotic factors, topography, distance to water etc.) were excluded as they may not have as much influence on where researchers locate their studies. Below we outline arguments for selecting these four key variables for this study.

‘Reptile species richness’ was derived from relevant geographic distribution data available through the work of Roll *et al*. (2017) and made accessible on the Global Assessment of Reptile Distributions (GARD) website (version 1.1, 2017). Each individual species distribution polygon was clipped to the Australian landmass boundary and then intersected with a 10 × 10 km grid cell (100 km^2^) fishnet layer to determine the number of species expected to occur in each cell. This resolution was selected to reflect (a) the wide variety of sources and, as evidenced by distances >1 km between vertices, global scale used for capturing species distribution layers^30^ and (b) differences in accuracy of study location coordinate recordings. Accuracy rating was determined by the number of decimal places used for the degree coordinates (decimal or DDD MM SS.S) when recording study locations. The predominant rating class covered a range of 1.11 km to 11.09 km.

The ‘human footprint’ layer was obtained from the Global Human Footprint Index layer developed by Venter *et al*. (2016). This layer is representative of human impacts at the landscape scale where the index is a number between 0 and 50, with 0 representing no human influence and 50 the maximum human influence. It is derived from eight, mostly correlated layers: roads, human population density, pasture lands, crop lands, railways, night-time lights, navigable waterways and built environments. For this reason, these stand-alone layers were not considered in isolation in our models. Standardised index values were available as a single layer at 1 km^2^ resolution^31^.

The ‘protected area’ layer was sourced from a national polygon layer published in 2016 under the Collaborative Australian Protected Areas Database (CAPAD) project by the Commonwealth Department of Environment and Energy^32^. Protected areas are defined as any area within the Australian protected area database and include all binding formal and informal reserves (i.e. national parks, reserves, indigenous protected areas, conservation covenants, etc.)^32^. This layer was converted into a binomial raster with each 100 km² cell having a value of one if more than 50% of the cell was covered by a PA polygon or zero if this condition was not met. Temporal variation in the establishment of individual protected areas was not included. Using a binomial raster also excluded consideration of differences in the level of PAs since this layer was primarily used as a surrogate for well-known biodiversity hotspots or areas of other high conservation values that have received statutory or administrative recognition over the past 50 years. Protected areas were also used as a proxy for threatened species where these networks support varying ranges for different taxonomic groups^33^. Researcher knowledge of the ‘existence of threatened species’ may have affected study location selection; however, this variable was excluded due to major and therefore complex changes in scientific knowledge as well as statutory instruments (e.g. variation is threat status classification systems among states), and therefore inventories, that defined threatened species and their communities.

**Figure 2:**
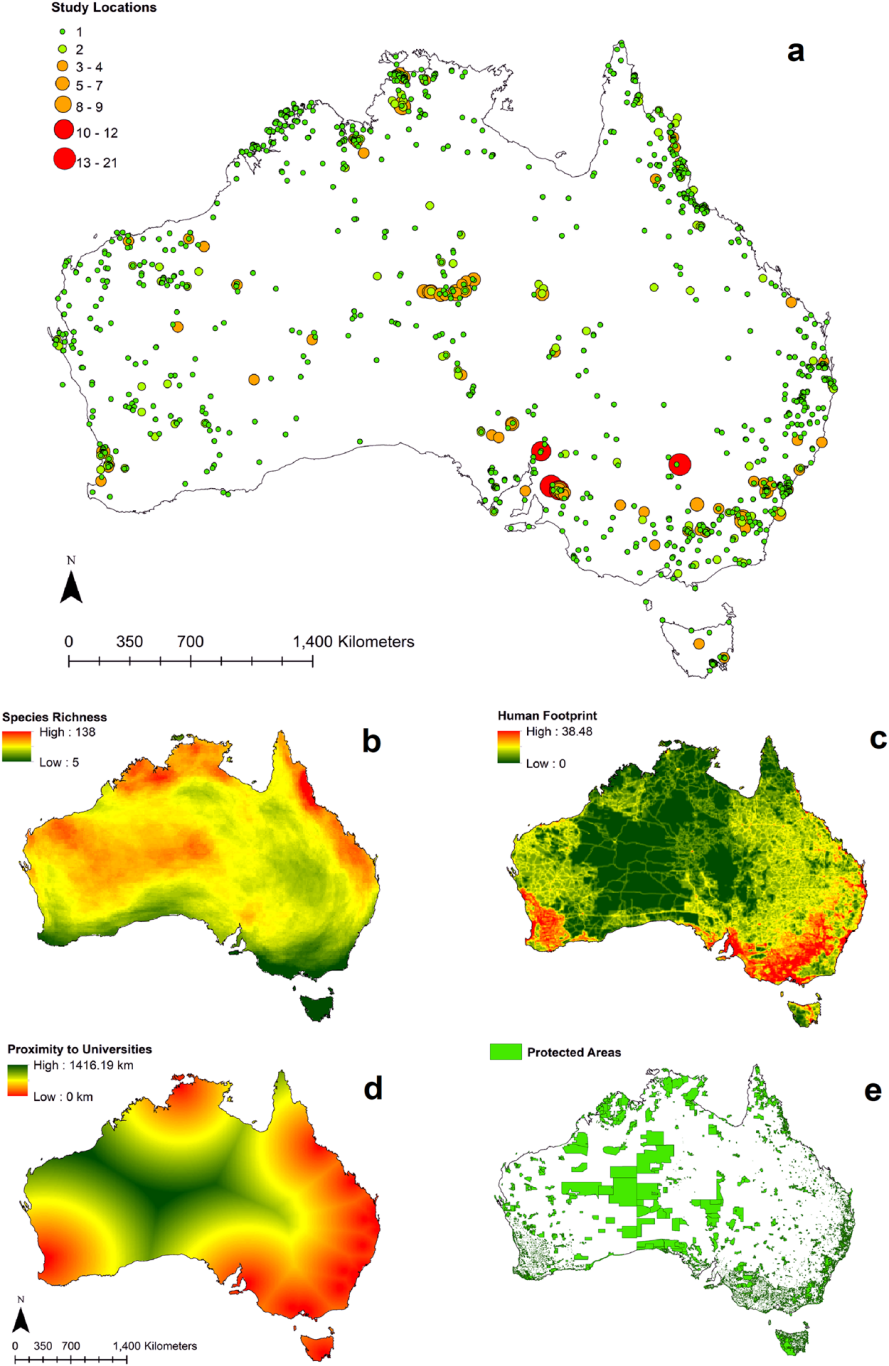
Map of Australia showing (**a**). Individual study locations with the number of study locations identified with larger markers (locations with 10 or more separate sites emphasised in red), medium markers (3–9 sites, orange) and small markers (1–2 sites, green), (**b)**. Species richness indicating high richness in red through to low richness in green, (**c)**. Proximity to universities showing close proximity in green and far proximity in red, (**d)**. Distribution of human footprint showing high densities in red and low densities in green, and, (**e)**. Protected areas shown in green. Maps were produced in ArcGIS 10.4™ (ESRI).

‘Proximity to universities’ was regarded as representing access to a critical mass of logistic and intellectual resources required for supporting field research and its subsequent authoritative analysis of samples. As indicated below (Fig. 2d), much of the Australian land mass has a low or even zero human footprint index, which imposes considerable challenges for undertaking field trips into these largely remote areas. Although most universities and relevant national or state research organisations have outpost research stations, these were still reliant on support from their principal hubs, i.e. the central location of their parent institutions, and therefore within a reasonable distance. Accordingly, the presence of a public or private university was considered as a suitable proxy for a centre of critical research mass required for undertaking field work. These locations also captured hubs of other research organisations in Australia such as state environmental agencies, NRM bodies and NGOs that were typically located in the same urban centres as universities.

Where they included the address of a major university campus, centroid coordinates of polygons from the Urban Centres and Localities layer of the Australian Bureau of Statistics for the 2011 population census^34^ were used for geolocating research hubs. Presence of more than one university or several research organisations was not given any extra weight. The distance to the closest university hub was calculated for each 10 km grid cell using the gDistance function in the rgeos package^35^ in R (v 3.5.1 R Core Development Team 2013).

We investigated the collinearity between all pairs of the four variables and found that all correlations were <0.6. We therefore included all variables in our model. All layers were resampled in ArcGIS 10.4 to a 10 km × 10 km (100 km^2^) resolution raster stack prior to running the Maxent model.

### Maxent model

The effect of the four predictors on study location (i.e. research effort response variable) was investigated using a SDM approach^26,27^ using open source Maxent software (ver. 3.4.1)^36^. We assigned a maximum of 10,000 background points. All variables were continuous with the exception of protected areas, which was binary. Default settings for features and regularization were selected. We used a 10-fold cross-validation test to determine the mean fit of the model, which gave an AUC value and standard deviation (limits included 0.5 meaning groups do not differ, and 1.0 meaning there is no overlap^37^ A jackknife test measured the importance of each predictor variable in the model, both collectively and independently. The model was fitted with all four predictor variables using linear and quadratic parameters, and logistic transformations.

We then used the resultant Maxent predicted study location raster to identify those regions within Australia that were distant from either universities or historical study locations while also having a low likelihood of a study taking place. Here we set a nominal threshold of <20% from the Maxent predicted raster. We converted the Maxent raster to a point layer in ArcGIS and then used the ‘near’ function to calculate the distance to points in a combined point feature layer of study locations and university city centroids. The resultant ‘cold spot’ layer was then converted back into a raster. We calculated the mean distance for all grid cells in 20% bins based on the Maxent predicted grid cell values.

## Results

A total of 1201 papers documented research into 387 reptile species across Australia. Geographic coordinates were missing from 46% of papers leaving 646 articles and 1631 individual study sites for spatial distribution analysis. Reported reptile study sites were primarily located in Western Australia (n = 408; 25%). New South Wales and Queensland each had 301 sites (18%), while the lowest number of sites was found in Tasmania (n = 37; 2%) (Fig. 2a). These patterns did not match relevant rankings in either total area, population size or the number and coverage of protected areas (Table 1). For example, reptile study effort in the Victoria was the most notable outlier having the second smallest landmass area, second highest resident population and the highest number of protected areas covering almost one sixth of the State’s total area but only the second lowest number of reptile study sites (n = 69; 4%) (Table 1). The number of research papers published increased over time with one paper in 1972 to 44 in 2016 (numbers decreased in 2017 (n = 11) due to an incomplete dataset). The maximum number published in a single year was 66 from both 2014 and 2015, and 51% of the papers have been published in the past 10 years.

**Table 1:** Summary values for key predictor variables in different state and territories in Australia.

Key variable parameters	Qld	NSW*	Vic	Tas	SA	WA	NT
Landmass (10^3^ km^2^)	2,030	1,121	354	123	1,310	3,121	1,508
Study sites	301	301	69	37	249	408	266
$\bar{X}$ nearest distance ± 1 SE (km)	14.8 ± 1.6	10.8 ± 1.3	19.7 ± 3.1	11.2 ± 4.8	10.0 ± 1.9	19.1 ± 1.4	9.9 ± 1.5
Study site density (study km^−2^)	0.00001	0.00027	0.00019	0.00030	0.00019	0.00013	0.00018
Sites per paper ± 1 SE	2.8 ± 0.7	2.5 ± 0.6	1.7 ± 0.2	2.7 ± 1.0	1. 7 ± 0.2	2.6 ± 0.8	4.5 ± 1.3
Species richness (100 km^−2^)							
Average	82.7	69.7	45.8	12.7	74.8	85.8	88.4
Max	138	98	76	17	99	122	122
Min	50	29	26	5	13	15	16
Human population 2016 (10^3^)	4,779	7,878	5,927	519	1,736	2,590	245
Population density (p km^−2^)	2.35	7.03	16.74	4.22	1.33	0.83	0.16
Percent of total population (%)	20.2	33.3	25.0	2.2	7.3	10.9	1.0
PAs	1,190	1,104	3,225	1,612	1,990	1,894	115
PAs total area (10^3^ km^2^)	160	79	52	29	296	588	340
PA coverage of landmass (%)	7.9	7.1	14.7	23.6	22.6	18.8	22.5
‘University locations’	6	7	2	1	1	1	1

NOTE: Qld = Queensland, NSW = New South Wales, Vic = Victoria, Tas = Tasmania, SA = South Australia, WA = Western Australia, NT = Northern Territory; * = includes the Australian Capital Territory (ACT).

Overlays of species distribution maps from the GARD dataset revealed highest reptile species richness for areas in northern Queensland (138 species per 100 km^2^) while this was lowest in Tasmania (Fig. 2b). Other areas with a higher than average richness of reptile species were identified along the middle of the continent’s northern coastline and its central landscapes towards the western coast (Fig. 2b).

Australia’s intense concentration of urbanisation within a handful of major cities was reflected by the highest values of the human footprint index for the greater Sydney, Melbourne, Brisbane, Perth and Adelaide regions. Larger areas of high development intensity were mapped for areas inland of the south-eastern and south-western coasts of Australia (Fig. 2c). These patterns were largely mirrored by proximity to universities with some additional locations in northern Queensland and the Northern Territory (Fig. 2d).

The georeferenced database of Australian protected areas (Fig. 2e, CAPAD, 2017) included 11,181 polygons with an average size of 138 (±1903) km^2^. Of all geolocated reptile study sites, 31% fell within protected areas capturing only 2% of the overall number of protected areas (Fig. 2a,e).

**Figure 3:**
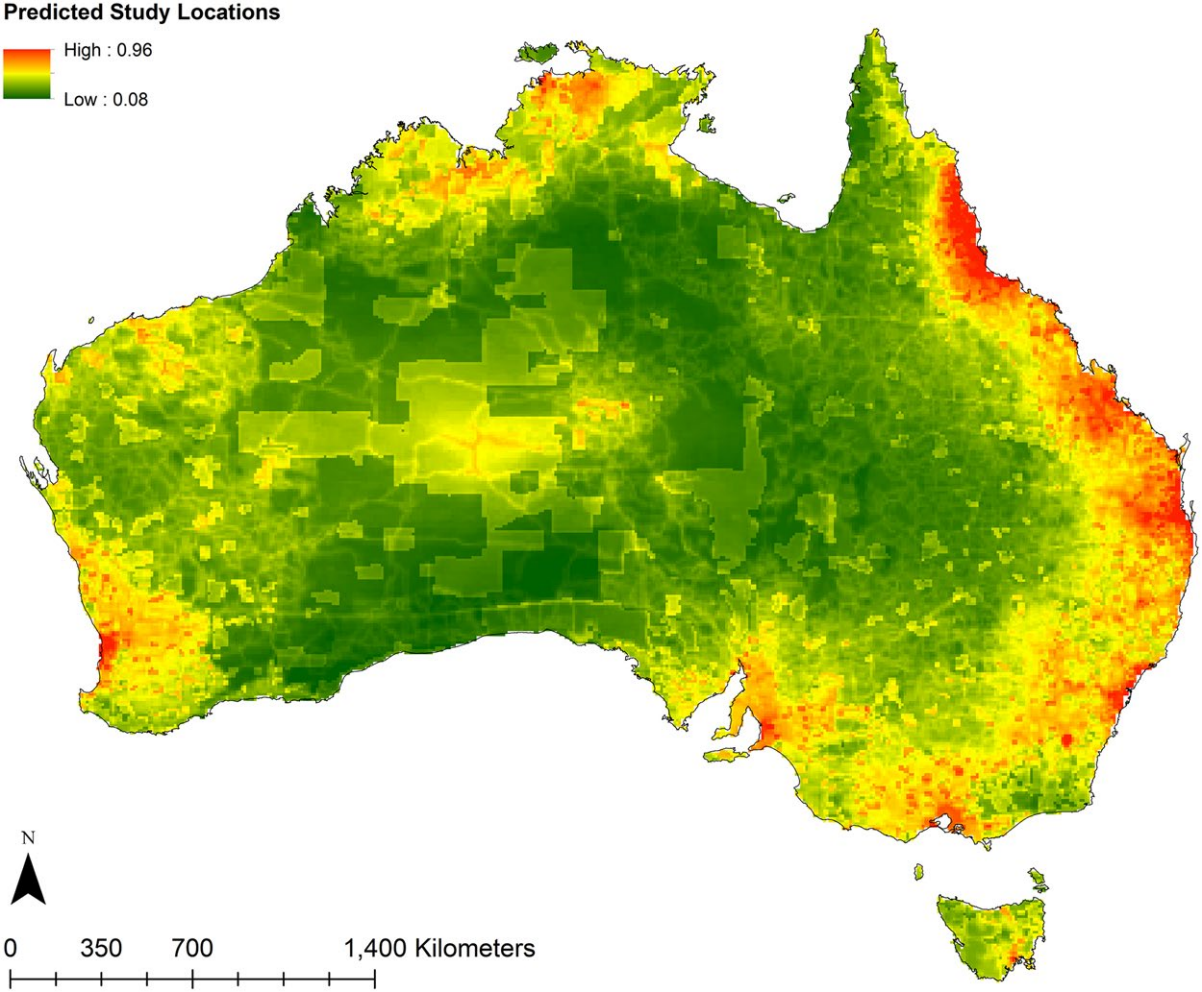
Model results showing the predicted study location preference based on current records using four variables (species richness, human footprint, proximity to universities and protected areas). Colour gradient indicates green as less likely to occur, through to red indicating high predictability of occurrence. Map was produced in ArcGIS 10.4™ (ESRI).

**Figure 4:**
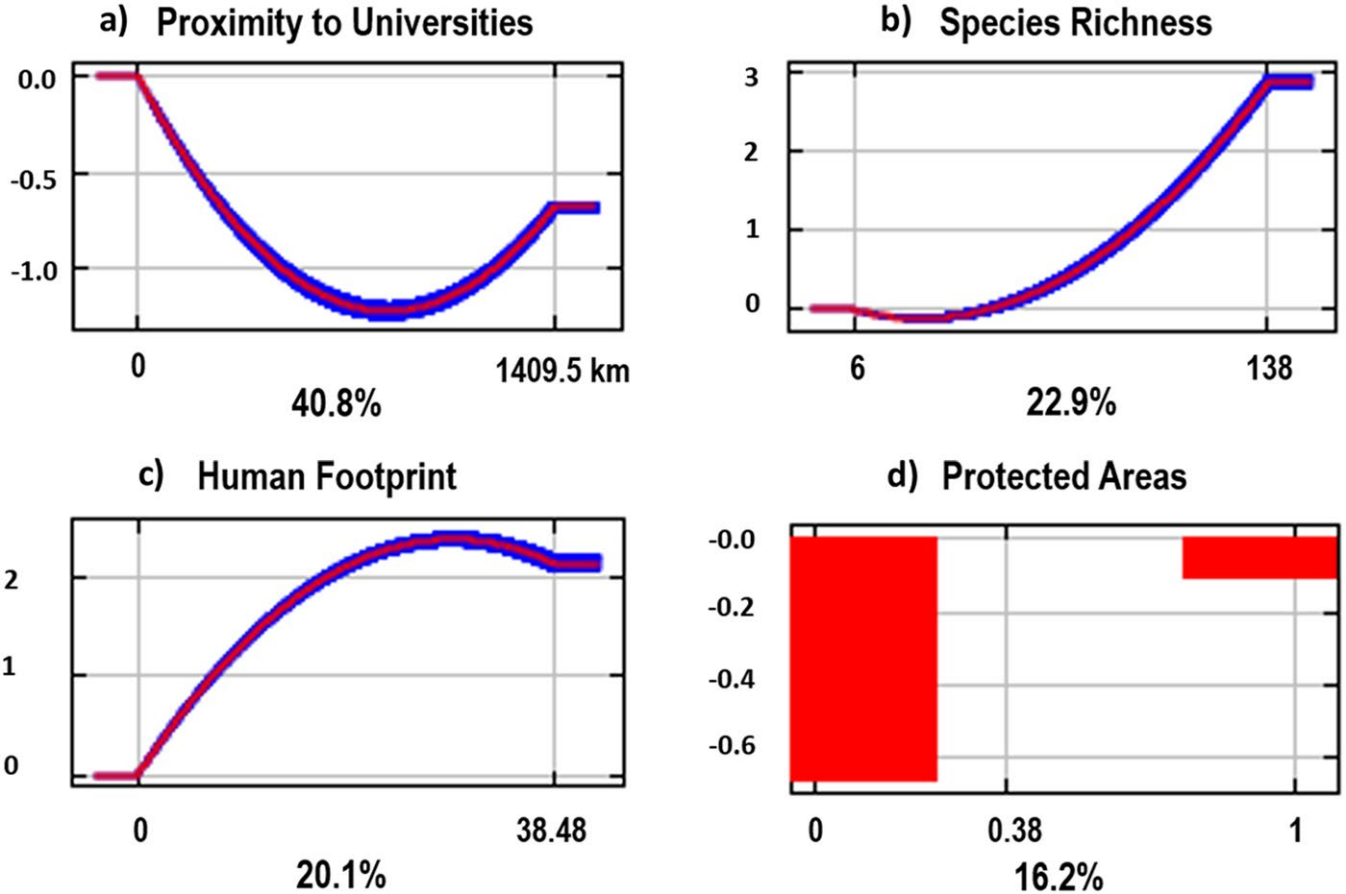
The response curves showing how each of the four variables, (**a**) proximity to universities; (**b**) species richness; (**c**) human footprint; (**d**) protected areas, affects the Maxent logistic prediction. Outputs have been averaged from 10-fold cross-validations. Due to protected areas being a categorical (0, 1) variable, the output was selected from one of the cross-validation runs for easier interpretation. Variable importance (%) shown below each graph. Y-axis is the probability of finding a reptile study site. Graphs were extracted from model results using the open source Maxent software (ver. 3.4.1)^36^.

**Figure 5:**
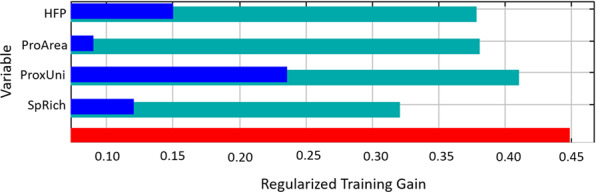
The jackknife test bar chart showing the importance of each variable when run independently. Light blue bars represent test without the variable, dark blue represents test with variable only and red represents all variables. Graph was extracted from model results using the open source Maxent software (ver. 3.4.1)^36^.

Based on study locations reported in the literature, the Maxent model revealed that proximity to universities (40.8%) contributed most to predicting reptile study locations (Figs. 3 and 4a). Species richness and human footprint contributed less to the model (22.9% and 20.1% respectively) (Figs. 3 and 4b,c), while protected areas contributed only 16.2% of the model (Figs. 3 and 4d). These results were reflected in the jackknife variable importance test, however species richness had less influence on the model prediction when considered independently (Fig. 5). A 10-fold cross-validation of test samples resulted in an average AUC of 0.757 (std dev = 0.035), meaning there is a 75.7% probability of correctly identifying reptile study locations across the continent based on the model predictors.

**Figure 6:**
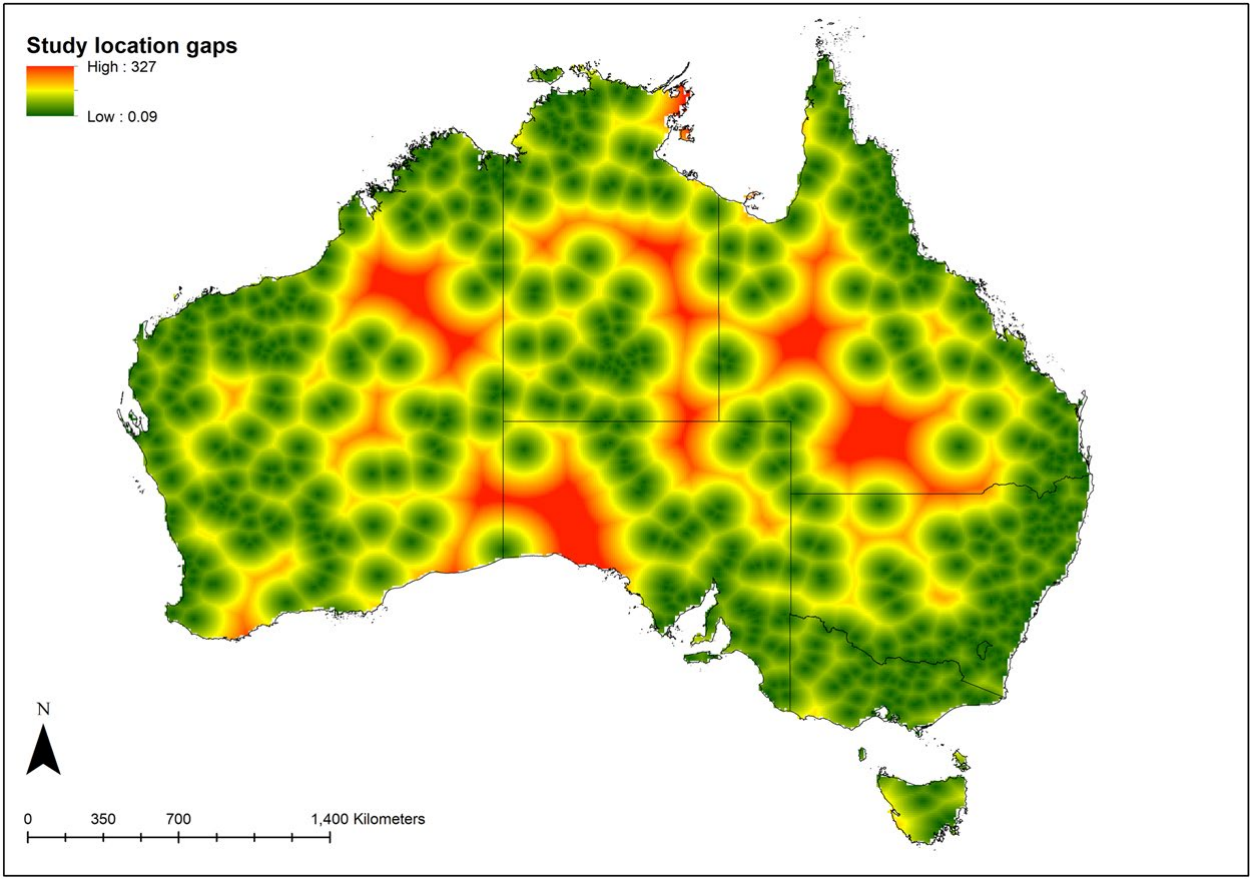
Representation of reptile research location ‘cold spots’ within Australia. The regions in red represent those that are most isolated in terms of their distance to historical locations as well as universities. Map was produced in ArcGIS 10.4™ (ESRI).

**Figure 7:**
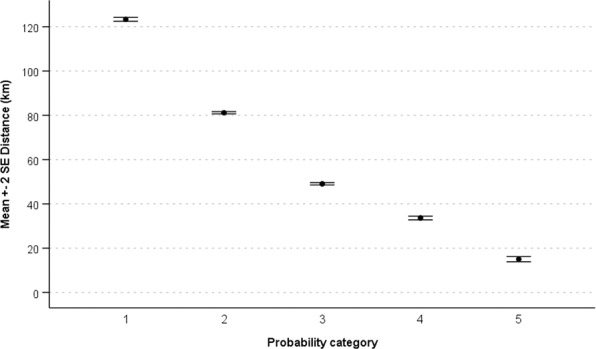
The mean distance (±SE) of the predicted Maxent study location output grid cells to historical study locations and universities combined. Grid cells with the lowest predicted likelihood of studies (Category 1 = <20%) were most isolated while those with the greatest predicted likelihood (Category 5 = >80%) were relatively accessible.

Study location ‘cold spots’ were identified in large parts of western Queensland, the eastern regions of the Northern Territory as well as the far north-eastern parts of Arnhem Land, western South Australia and north-eastern Western Australia (Fig. 6). These regions were typically between 200-330 km away from either universities or other study locations. There was a significant different in the distance from either universities or historical study locations for all Maxent predicted output values (20% bin categories) (F = 6149.8, d.f. = 4,86495, P < 0.0001) (Fig. 7), highlighting areas that are potentially oversampled. Grid cells with the lowest study location predictability were more isolated (i.e. greatest neighbourhood distances) (mean distance = 123.3 ± 0.44 km, n = 21761) that those with the highest predictability (mean distance = 15 ± 0.59 km, n = 447) (Fig. 7).

## Discussion

Understanding geographical biases in ecological research is becoming increasingly important for determining what may be impacting on biodiversity^2,4,5^. This is the first continent-wide analysis of spatial bias associated with Australian terrestrial reptile ecological research. Research study locations appear to be most influenced by proximity to universities, followed by species richness and human footprint, while the weakest influence was from protected areas. The combination of social and environmental drivers in predicting reptile study locations is not unexpected, as similar combinations were identified as predictors of conservation easements in the United States^28^. The convenience and accessibility of survey sites can be a key driver in ecological research location selection as noted in studies on African mammals where bias was evident towards the proximity to universities and museums^38^, with the human population index influencing study densities in Australian flora records^20^, and with roadside collection biases in herbaria collections^8^. Additionally, the ease of access has been associated with increases in the density of study locations, particularly for koala records in Australia^39^, and studies of birds in Africa^4^. The importance of ‘proximity to universities’ does not imply that research studies are more likely to be undertaken on university grounds per se, but that study locations have a greater likelihood of being close to these institutions or associated urban centres. Isolated sampling within and close to urban hotspots could not only lead to biased representation of species ecology, but erroneous estimates of anthropological impacts^19^. Considering the importance placed on accurate spatial data to inform conservation decision-making^40,41^, there is a potential for input data to be biased through sampling and this could lead to weaker conservation outcomes.

Although the model predicted that proximity to universities had the greatest influence on study location preference, the results also indicated higher study location density in areas of higher reptile species richness. Regions of high diversity are often targeted by researchers due to the increased chance of observations and the known success of previous recordings^19,42,43^. While research in such highly diverse areas has been vital in describing new species over the past several years^10^, prioritising such areas could lead to other regions being underrepresented in studies^20^. Our model provides an opportunity for researchers to identify future study locations that complement existing data and provide greater representation of all regions, while also highlighting areas that may be prone to oversampling. Of course, access in remote areas will continue to be a challenge and it is not surprising that research activity was associated with the human activity index. Researchers typically utilise road networks to access study areas unless the species of interest can also be recorded through airborne observations (e.g. waterbirds, large mammals)^44,45^. While future surveys might still rely heavily on road networks for access to regions, new technologies such as drones provide an opportunity to survey some large species in remote areas^44^.

Protected areas alone are unable to maintain or conserve global biodiversity, emphasising the importance in thoroughly understanding the impact of threatening processes, and how to minimise such pressures^46^. Protected areas not only require an understanding from an ecosystem perspective, but finer scale interpretation of species-specific inhabitants is needed to provide sound planning for conservation strategies^47^. Our review found that study locations were generally not greatly influenced by protected areas, despite previous studies indicating an overrepresentation of ecological research in these areas^5,20^. Our model is also likely to overestimate the number of studies located in protected areas as no temporal correction was made for studies that were completed in areas prior to any protected area designation. This highlights that for reptiles at least, research effort has been more widespread in its coverage.

Our analysis also identified those areas where there has been less research effort to date based on the Maxent model output as well as a subsequent distance-based assessment of each grid cell. These research ‘cold spots’ that comprise some ~5.6% of the Australian landscape are located in most Australian states and territories with only areas in Victoria, the ACT and Tasmania being adequately covered. Directing research effort to these ‘cold spots’ will assist in filling knowledge gaps for Australian reptile communities.

While it is important to understand the impacts from human disturbance and associated threats on ecological systems and biodiversity^2,21^, there is also a need to identify gaps in our knowledge that may impact on conservation decision making and landscape management. In this fine-grain continental analysis we have shown that social elements largely determine where research is conducted. However, our analysis also only reveals a static interpretation of the spatial distribution of reptile research. It may still be necessary to improve our understanding of the dynamism within fragmented landscapes amidst such regions that could provide vital information on the ecological status of reptiles. For instance, areas comprised mainly of agricultural lands can undergo changes in landscape components on a regular basis^48^. The mosaic landscapes can provide important habitats for many reptile species, and due to the ongoing disturbances in these regions, further ecological research is needed to gain a broader understanding of the overall biodiversity status^5^. We suggest that to successfully assess the ecological status of Australian reptiles, institutions and funding agencies need to support researchers in reprioritising study locations based on current spatial deficiencies. This will enable an unbiased measure of pressures and threatening processes across multiple scales and deliver stronger governance for their protection and conservation.

## Supplementary information

Supplementary Information.
