# Supplementary material for: Location biases in ecological research on Australian terrestrial reptiles
Source: Sci Rep. 2020 Jun 16;10:9691. doi: 10.1038/s41598-020-66719-x (PMC7298028; doi:10.1038/s41598-020-66719-x)
Supplement: Supplementary file 1 — Supplementary Information. [file 41598_2020_66719_MOESM1_ESM.pdf]

# Location biases in ecological research on Australian terrestrial reptiles

*Renee Louise Piccolo[1,4] (BSc Honours), email: r.piccolo@griffith.edu.au

Jan Warnken[1,3] (PhD), email: j.warnken@griffith.edu.au

Alienor Louise Marie Chauvenet[1,2] (PhD), email: a.chauvenet@griffith.edu.au

James Guy Castley[1,2] (PhD), email: g.castley@griffith.edu.au

1) School of Environment and Science, Griffith University, Gold Coast campus, Australia, 4222
2) Environmental Futures Research Institute, Griffith University, Gold Coast campus, Australia, 4222
3) Australian Rivers Institute, Griffith University, Gold Coast campus, Australia, 4222
4) Corresponding author

## Supporting Information

### Piccolo et al.

**S1.** Search criteria used primary keywords separated using the OR operator. These were combined, using the AND operator, with secondary keywords and filtered the excluded words.

| Primary Keywords | Secondary Keywords | | Terms Excluded |
|---|---|---|---|
| "Australia*" | "Reptil*" | "Lizard*" | "Sea" |
| "Tasmania*" | "Herpet*" | "Testudin*" | "Marine*" |
| "Queensland*" | "Crocod*" | "Turtle*" | "Ocean*" |
| "Victoria*" | "Lizard*" | "Tortoise*" | "Frog*" |
| "Territor*" | "Skink*" | "Snake*" | "Amphib*" |
| "Northern Territory*" | "Scinc*" | "Elapid*" | |
| "New South Wales*" | "Pygopod*" | "Colubrid*" | |
| | "Agamid*" | "Python*" | |
| | "Dragon*" | "Typhlopid*" | |
| | "Varan*" | | |

**S2**. Web of Science categories included in the search.

| Web of Science Categories | | |
|---|---|---|
| Agriculture | Evolutionary Biology | Physiology |
| Anatomy Morphology | Fisheries | Plant Sciences |
| Behavioural Sciences | Forestry | Reproductive Biology |
| Biodiversity Conservation | Geology | Science Technology |
| Biology | Marine Freshwater Biology | Urban Studies |
| Developmental Biology | Multidisciplinary Sciences | Veterinary Sciences |
| Ecology | Palaeontology | Virology |
| Entomology | Parasitology | Water Resources |
| Environmental Sciences | Physical Geography | Zoology |
| Environmental Studies | | |

**S3**. List of research focus categories and number of studies.

| **Research Focus Categories** | | | |
|---|---|---|---|
| Behavioural | No. of Studies | Ecological | No. of Studies |
| Behavioural | 165 | Distribution/Geographic | 116 |
| Movements/Dispersal | 154 | Temperature | 115 |
| Mating/Sexual Dimorphism | 106 | Abundance | 100 |
| Fire Response | 56 | Diet | 100 |
| Foraging | 51 | Predator | 87 |
| Weather/Climatic Responses | 42 | Alien/Invasive | 80 |
| Defence | 22 | Seasonal | 73 |
| Territoriality | 13 | Population | 69 |
| | | Richness | 59 |
| Physiological/Molecular/Taxonomic | | Prey | 54 |
| Reproduction/Hybridisation | 207 | Parasitism | 39 |
| Genetics/DNA Sequencing/Blood Analysis | 200 | Microhabitat | 38 |
| Nests/Eggs | 129 | Resources (water etc) | 34 |
| Morphology | 111 | Social/Species Interaction | 33 |
| Body Condition/Size | 74 | Human Impact | 30 |
| Thermoregulation | 49 | Diversity | 28 |
| Physiology | 44 | Trapping/Tracking/Survey Methods | 20 |
| Phylogeny | 39 | Climate Change | 18 |
| Taxonomic/Nomenclature | 32 | Competition | 17 |
| Toxicity/Venom | 22 | Adaptation | 16 |
| Scats/Tracks | 12 | Pollution | 14 |
| Palaeontology/Fossil/Dating | 10 | Evolution | 13 |
| Isotopes | 3 | Community | 10 |
| | | Harvesting/Hunting | 9 |
| Structural | | Translocation/Relocation/Reintroduction | 9 |
| Habitat Specificity/Niches | 212 | Presence/Absence | 4 |
| Vegetation | 78 | Disease/Virus | 4 |
| Agriculture/Cultivation/Grazing | 60 | Endemism | 3 |
| Fragmentation | 54 | Recreation & Tourism | 2 |
| Disturbance | 39 | Rehabilitation/Reintroduction | 2 |
| Urbanisation | 31 | | |
| Restoration/Revegetation | 26 | | |
| Mining/Infrastructure | 18 | TOTAL OCCURRENCE IN STUDIES | |
| Substrate | 12 | Behavioural | 609 |
| Deforestation/Land Clearing | 11 | Physiological/Molecular/Taxonomic | 932 |
| Forestation/Plantations | 7 | Structural | 556 |
| Roads/Crossings | 4 | Ecological | 1196 |
| Corridors | 4 | | |
